# 'No Matter How Harsh, We Are Alive': Coping Strategies of Rural–Urban Migrant Homeless Older People in Ethiopia

**DOI:** 10.1111/hsc.13848

**Published:** 2022-05-20

**Authors:** Getachew Gebeyaw, Messay Gebremariam Kotecho, Margaret E. Adamek

**Affiliations:** ^1^ University of Gondar, Department of Social Work Gondar Ethiopia; ^2^ School of Social Work Addis Ababa University, University of Johannesburg Addis Ababa Ethiopia; ^3^ Indiana University School of Social Work Indianapolis Indiana USA

**Keywords:** coping strategies, ethiopia, homeless older persons, homelessness

## Abstract

The prevalence of homelessness among older adults in Ethiopia is growing. While prior studies examined the push factors and challenges of homeless elders, little is known about how older people cope with homelessness in a context of limited ageing services. This study explored the coping strategies of homeless older people in Kobo Town, Ethiopia. Purposive sampling was used to identify 10 homeless older people and four key informants. Thematic analysis was used to analyse the data collected through in‐depth interviews. To cope with the challenges faced on the street, homeless older people used various strategies including begging, holy water, drying leftover food, using river water for hygiene and sanitation, sleeping in church compounds, and creating their own social networks. Despite their best efforts, elders' coping strategies could not overcome the barriers imposed by their resource‐poor environment. The findings call attention to the urgent need for a national response to elders' unmet needs including an income support programme and multifaceted support services for older adults in Ethiopia.

**What is known about this topic?**

- The number and proportion of older adults in Sub‐Saharan Africa are growing.
- As a result of migration and modernization, traditional family support of older adults in Sub‐Saharan Africa has been waning for decades.
- The lack of income protection and support services for older people in Sub‐Saharan Africa contributes to the growing problem of homelessness.

**What this paper adds**

- While the factors contributing to homelessness among older adults are documented, this paper provides older adults' perspective on how they cope with homelessness.
- In the absence of family and government support, homeless older adults rely heavily on begging to meet their survival needs.
- Despite several active coping strategies, older adults' efforts do not lead to exiting homelessness.

## 1 INTRODUCTION

By 2050 over 80% of the world's older population will be living in developing nations (United Nations, 2019). This growth is occurring in nations that are the least prepared to support an ageing population. Sub Saharan Africa is projected to have the second highest proportional increase (over 200%) in the older population by 2050 (United Nations, 2019), yet this world region lacks both social protection and infrastructure of health and social services to support an ageing population. In the past, when extended families traditionally cared for ageing family members, the absence of income support and ageing services was not so problematic. However, major societal trends taking place in Sub Saharan Africa, such as migration, urbanisation and the HIV pandemic have altered the nature and extent of family support for older adults (Adamek et al., 2020; Kotecho et al., 2022). While an estimated 1.5 million children in Ethiopia were orphaned by the HIV/AIDS crisis in the 1980s and 1990s, the nation has achieved the 75% mortality reduction target due to HIV set for 2020 (Mirkuzie et al., 2021) with an overall adult prevalence of HIV of <1% (Adal, 2019). Though AIDS has progressed from a fatal disease to a chronic illness to be managed, the impact on family structures has been long‐lasting with many grandparents becoming primary caregivers of their grandchildren (Abebe & Aase, 2007). Instead of having adult children to rely on for support in their old age, the AIDS crisis weakened family supports for elders (Adamek et al., 2020). Further decline of family support for older adults is occurring due to the growing migration of young people from mainly rural areas in Ethiopia—where it has become normative for most families to have their young adults migrate to the Middle East (Adamek et al., 2020).

The growing number and proportion of older adults in Sub‐Saharan Africa, alongside the lack of ageing infrastructure and social protection and waning family support, is setting the stage for a growing number of destitute and homeless older adults.

While homelessness among older adults occurs globally, most of our understanding of this phenomenon stems from studies in the Global North. Yet, the factors contributing to homelessness among older adults in the Global North and the services available to them are very different. For example, in Canada (Hwang et al., 2009), the US (Brown et al., 2012), Australia (Lipmann, 2009) and throughout Europe (Busch‐Geertsema et al., 2010), a range of services and shelters are available in major cities to provide refuge and support for homeless individuals, including older adults. An array of both governmental and non‐profit organisations offer a safety net for older adults at risk of homelessness. Organisations like Catholic Charities (2018) provide specialised services and have developed best practices in meeting the needs of homeless elders. Given the vastly different context of older adults in nations lacking such support systems, studies are needed to document and understand the unique context and experience of ageing and of homelessness in Global South nations.

As noted above, in many Sub‐Saharan nations including Ethiopia, younger people migrate to urban areas in search of education and/or employment (Mberu, 2006), increasingly leaving older family members behind in rural areas. Indeed, internal migration has become a common livelihood strategy in Ethiopia (Kassegn & Endris, 2020). In a context of food insecurity, waning family support, uncertain climate conditions for farming and little no social protection, rural older adults themselves have increasingly migrated from rural areas in Ethiopia to urban centres. In a study of older adults' well‐being in rural Ethiopia, those who were no longer able to physically work to support themselves sometimes became victims of abuse or neglect (Chane & Adamek, 2015). The difficult context of ageing in Ethiopia is likely a major contributor to the relatively low life expectancy at birth (66.95 years)(World Data Atlas, 2020).

An assessment of older adults' needs in Ethiopia by HelpAge International (2011) found that unreliable sources of income, a lack of varied livelihood possibilities and restricted access to social and health services make it difficult for older Ethiopians to achieve household security. Reliance on a single source of income does not allow older people to maintain a stable income. For survival, a combination of livelihood methods is required, including working, seeking NGO assistance, receiving donations from children and other family members and begging. In both urban and rural locations, a major concern for older people is food insecurity (Hailu, 2020). During a food crisis, older adults' coping techniques include lowering food consumption, going without food for an entire day or night, opting for lower‐quality food or even substituting food with wild plants and sending children to live with strangers (HelpAge International, 2013). In addition, older people are more likely to feel isolated and uninvolved in their communities. Mirkena et al. (2018) documented a high rate of depression (over 40%) among residents aged 60 and over in Ambo Town, a small town in the Oromia region west of the capital city of Addis Ababa. Retired older adults in the Ambo study were four times as likely to show signs of depression. These rates are at least twice the rate reported by WHO (2017) who estimated that between 10 and 20% of older adults globally experience depression.

Extensive poverty among older adults in Sub‐Saharan Africa is contributing to the influx of rural older adults into urban areas in search of employment or other means of support (Adjaye‐Gbewonyo et al., 2020). While some impoverished older adults in Africa migrate from urban to rural areas (Adewale, 2005), family breakdown in rural areas serves as a significant push factor for rural older adults to migrate to cities (Abeje, 2021). Yet, in the absence of public income support for older adults and in light of persistent age discrimination, a significant portion of older adults who migrate to urban areas end up homeless (HelpAge International, 2013). Although a few studies have examined the plight of homeless older adults in other Sub‐Saharan nations such as Nigeria (Geyer, 2021) and South Africa (Hellandendu, 2014), the struggles of homeless older adults in Ethiopia have received little attention. Likewise, while there has been some attention to the push factors contributing to the growing problem of homelessness in the continent (Makiwane et al., 2010; Speak, 2020), less attention has been given to older adults' responses to homelessness. Thus, to enhance our understanding of responses to homelessness, this study aimed to explore the coping strategies employed by rural–urban migrant homeless older adults in Ethiopia.

## 2 MATERIALS AND METHODS

### 2.1 Design and sampling

Given the lack of studies that have documented the situation of homeless older adults in Ethiopia, we chose a descriptive multiple‐case study design. This approach involves in‐depth study of an issue through one or more cases within a bounded system (Creswell, 2007). Multiple‐ case studies allow researchers to analyse the data within each situation and across different cases. The evidence generated from a multiple‐case study is considered strong and reliable (Gustafsson, 2017).

Descriptive studies provide a picture of particular details and relationships within a social setting with the aim of accurately representing the scenario (Krueger & Neuman, 2006). According to Creswell (2007), in choosing a qualitative design researchers must decide on the central assumption or perspective of the study. In this case, the researchers assumed that the older adults were the best source of information on their experiences of coping with homelessness.

The study employed purposive sampling of homeless elders, Labour and Social Affairs officials and Elders Association leaders. Purposive sampling is relevant in a circumstance where the researcher selects what he or she believes is a typical sample based on specialised knowledge or selection criteria in the study sites (Walliman, 2006).

The study included 10 homeless older individuals over the age of 60, two representatives from older people's organisations, and two specialists from the Labour and Social Affairs office for a total of 14 participants. The criterion of data saturation was used in deciding the number of homeless older adults to include as participants. Thus, the sample size was determined by the point in data collection when new data no longer provides any further insights in addressing the study objectives (Mack et al., 2005; Suri, 2011).

### 2.2 Data collection

The data collection methods included in‐depth interviews, key informant interviews, observation and document review. Following Yin (2003), observation occurred during the field visit. For 1 month, the first author observed the sleeping places and materials used by older adults, the food they ate, the location they used for sanitation and hygiene and the daily activities of homeless elders. For triangulation purposes, field notes were taken throughout the data collection process. The data collection and analysis occurred simultaneously, with the observation data used to verify the narrative data from the in‐depth interviews. For instance, when elders shared about their coping strategies such as using holy water, leftover foods, and begging, the researcher directly observed the older adults using these approaches in their daily living.

Over the course of a month, 10 older persons who had been homeless for a year or more participated in in‐depth interviews spanning 35 to 85 min. The interviews were conducted in Amharic, Ethiopia's official language, and were subsequently transcribed and translated into English. The participants chose the location of the interview, which included cemeteries, church compounds and areas near mosques. A semi‐structured interview guide was used to document the perspectives of older persons regarding their experiences of homelessness. Probing was used to obtain detailed information from the participants regarding their coping mechanisms while living on the street. Key informants were asked about the coping techniques elders use to cope with living as a homeless person.

Data transcription, translation and analysis were conducted simultaneously, thus, data analysis and collection occurred iteratively until we reached a point of data saturation. When additional data collection does not yield new information, and no new codes are generated, data saturation has occurred (Fusch & Ness, 2015). Thus, we used the interview guide and probes to conduct a comprehensive interview with the first study participant. Second, while the researcher was in the field, we proceeded with transcription and translation for purposes of member checking with participants and generating initial codes. Third, the researchers identified contrast ideas and concepts to probe in subsequent interviews. Fourth, data from additional participants were used to further build the themes and consider the fit of the data with the emerging themes. Fifth, in accordance with the study objectives, the researchers considered the substance of the developing ideas and whether they required further unique theming and data. Finally, upon noting repetition of responses in the last interview, the researcher decided to stop further interviews.

Finally, the first author reviewed written documents from national, regional and local government offices relating to older people in general and homeless older people in particular, including the Social Protection Policy, the National Plan of Action on Older Persons and relevant reports from the Ministry of Labour and Social Affairs, as well as local government reports. To ensure the study's quality, data from the document review was used to triangulate the narrative data gathered from the in‐depth interviews, key informant interviews and observations. The national, regional (Amhara) and woreda (local) level reports of the country confirmed that homeless older people face various challenges on the street. The methods older adults use to cope with homelessness such as begging and using river water for hygiene and sanitation were likewise noted in various government reports.

### 2.3 Ethical procedures

To protect the participants' rights throughout the study, the researchers followed all ethical procedures. Ethical clearances were obtained from Addis Ababa University (AAU) and the local woreda administration bureau. The first author presented a letter of cooperation from the AAU School of Social Work describing the nature and purpose of the study to officials at the Kobo Town Labour and Social Affairs Office. The government officials gave consent for data to be collected from homeless older adults in the area.

An informed consent form was developed indicating the aim of the study, the procedures for preserving confidentiality, the relevance and eligibility of participation, and the right to skip questions or withdraw from the study at any time. The first author approached the participants on the street to introduce the study and request their participation. Participants gave permission for the interviews to be audio‐recorded. Pseudonyms were used in the presentation of findings to preserve participants' confidentiality. After data analysis was completed, the researcher discarded the original recorded raw data which collected from the study participants.

At the close of the study, the first author received 10,000 ETB from the University of Victoria in Canada to benefit homeless older adults in the study area. The funds were used to provide 3 days of training to religious and community leaders, health workers, para‐social workers, gender experts, Labour and Social Affair officials, high school teachers and youth volunteers in Kobo Town. The aim of the training was to show the extent of challenges that older people face on the street, to organise a task force to support homeless older people, to create advanced institutional support systems and to mobilise resources for a long‐lasting solution. As a result, selected homeless older people were offered a congregate residence. For those elders who chose not to move into an institutional setting, the youth volunteers were organised to mobilise resources to provide clothes and food on a regular basis. Finally, all of the research participants were invited to a dinner programme and briefed about the new taskforce organised to empower older people and to welcome their participation in all areas of the problem‐solving process.

### 2.4 Data analysis

The interview guides were prepared in Amharic, the official language of Ethiopia. After each interview, the first author, with oversight by the second author, transcribed the participants' narratives into written form in Amharic. Next, the transcribed data were translated into English by the first author. The third step involved back‐translating the English data into Amharic by a peer researcher to check for the accuracy of the translation. Finally, the researchers moved to the interpretation process using the English version of the dataset.

While there is no one right way to analyse qualitative data (Lacey & Luff, 2009), much qualitative analysis falls under the general umbrella of thematic analysis. Member checking occurred iteratively during data collection and analysis. After each interview was transcribed in the field, the first author read the transcript aloud to each participant to validate the data. To develop themes and patterns, we followed Braun and Clarke's (2006) six steps of familiarisation with the data, generating initial codes, searching for themes, assessing themes, defining and identifying themes and producing the report.

### 2.5 Study Participants

Of the 10 older adults interviewed for this study, seven were men. Either through divorce or being widowed, all 10 participants had lost their spouses. Respondents ranged in age from 62 to 85 with a mean age of 72.4. All were followers of Orthodox Christianity and none had finished school. Two respondents had no children and the other eight respondents had from 1 to 7 children. Respondents had been homeless for an average of 4 years (2 to 11 years).

## 3 RESULTS

### 3.1 Coping strategies employed by homeless older people

In the face of different challenges of living on the street and around the churches, study participants employed various coping strategies to minimise their challenges. The homeless older people in this study used six types of coping strategies: begging, holy water, using the river for hygiene and sanitation, preferring to live around the churches, drying leftover food for future use and creating a social network among homeless older people.

### 3.2 Begging

Among the coping strategies that homeless older people employed, begging was the major source of income used to minimise their challenges. Throughout the in‐depth and key informant interviews, all three types of study participants revealed that begging is the primary and often only option that homeless older people used because their health problems hinder them from fulfilling their own basic needs through employment. The study participants beg for different things such as (1) food, (2) drinking water, (3) clothes, (4) money to buy food and medicine, (5) sleeping materials and (6) tela (local drink).

Additionally, the observational data revealed that older people beg on the street, at churches and in various locations throughout town. When there are celebrations in town, homeless older people gather to get food and drink tela, according to participant Habtam (85 years old): ‘*Even though life on the street is horrible, begging saves my life because all of the food I eat, clothes I wear, and water I drink come from begging’*. Abebe, a 75‐year‐old homeless man, explained:

I was frequently sick at nearby churches for the last five years, but I was able to cover the cost of my medication by begging for money on the street. However, I cannot guarantee that I will be able to cover my medicine costs on a regular basis.

### 3.3 Holy water

The health concerns of the older individuals in this study are exacerbated by their homelessness, a lack of affordable medication and the absence of access to free medical care. Due to a lack of resources, participants frequently used holy water to treat their health problems. When an older adult became ill, they prefer to drink holy water because they believe it will help them recover their health. Some homeless older individuals visited various churches outside of Kobo town in order to obtain holy water for their health issues. Homeless older individuals said they use holy water when they cannot afford needed medications. Because of their strong spiritual convictions, the older adults indicated that holy water was used to treat their health problems. In addition, another remedy the homeless older adults was used *‘Fel Woha’* (naturally hot spring water). When ‘*fel woha’* was available somewhere other than near churches, the homeless older people did not see it as holy water. When the ‘*fel woha’* is found near churches outside of the town, however, people see it as holy water and as God's blessing. Mengesha, a 70‐year‐old man who has been homeless for 3 years, relied on holy water to help him with his health problems. As he explained,

It is difficult for me to obtain funds to purchase medication. Instead, I've been using holy water to help me recover from my illness. God's pure blessed water is known as holy water. I thanked God for the valuable gift because whenever I use holy water to treat my health problems, I am always cured.

### 3.4 Use of river water for hygiene and sanitation

Using river water is one of the coping methods used by homeless older adults to deal with their hygiene and sanitation issues. Because they do not have easy access to clean water for washing their clothes and bodies, the homeless older adults in this study used the river for hygiene and sanitation. Despite the distance between where they slept in town and the river outside of town, the homeless older adults saw it as a means of coping with their difficulty. Older homeless persons have no other option but to use the river to solve their hygiene and sanitation issues. As a result, elders travel a long distance to access the river water for hygiene and sanitation purposes. Habtam, an 85‐year‐old woman, shared:

Despite the fact that the river is a long way from where I live, I've been using river water for hygiene and sanitation since the day I became homeless. However, because of my weak muscle strength, I am unable to travel on a regular basis for the sake of hygiene and sanitation, which is another contributing factor to my health problems.

According to the key informants, some city residents let homeless older adults wash their clothes in their private water. However, this does not work for everyone, and the bulk of community members are opposed to allowing water to be used by homeless elders for the hygiene and sanitation. Community members occasionally provide a bottle of water to homeless elderly for drinking purposes. According to the leader of the Elders' Association, accessing the river for hygiene and sanitation purposes is problematic for homeless older adults with mobility restrictions.

### 3.5 Living around religious institutions

As a coping mechanism, homeless elderly persons who took part in the in‐depth interview preferred to live near churches rather than other settings. They chose to stay near the churches because they believe it is a safer environment for them than sleeping on the street, on the veranda, or in places not designed for human habitation. Furthermore, homeless older adults said that when they sleep and live near churches because they have access to the people who regularly come to the churches for prayer and congregation. During the day, homeless older adults beg in various locations throughout the city and community, but at night, they congregate near the churches to sleep and communicate with their homeless peers.

A growing number of homeless older people sleep in the town's many churches. According to documents from Kobo Town's Labour and Social Affairs Office, there is a high concentration of homeless older persons in several churches. Because of the safety, access to food and money, social contact among the homeless and access to holy water, older homeless persons prefer residing near churches rather than alternative sleeping spots. The homeless older adults who live near churches have easy access to religious teachings that reinforce their patience and acceptance of the circumstances they find themselves in on the street, such as ‘God created everything for a reason’. Their enduring faith aids them in reducing despair and loneliness.

‘*No matter how harsh, we are alive*,’ said Belay, a 71‐year‐old homeless man who has been living on the streets for the past 4 years. ‘*As a homeless person, I've been sleeping in different religious institutions throughout town to get my daily necessities and to be protected from abusers and hyenas’*.

### 3.6 Drying leftover food for future use

When older people begged for food, they sometimes gathered a large amount of leftover food that was not fit for daily consumption. As a result, homeless older people sometimes preserve food by drying it in the sun. The dried food, known as ‘Koshero’, is saved for future use. According to the researcher's observations, homeless older people preserve portions of dried leftover foods in a sack or smaller bags for future use. Thus, homeless older adults use dried leftover food as a coping method to address their food insecurity. Furthermore, homeless older adults reported that they had trouble obtaining sufficient quantities of leftover items through begging during the fasting period. In times of food shortages, they rely on their dried leftover foods as a coping technique for everyday intake.

According to Belaynesh, a 68‐year‐old woman, ‘*During fasting and the rainy season, we had a food restriction. As a result, we gathered foods, dried them, and stored them for future consumption as a recuperation mechanism’*. Nigatu, an 85‐year‐old participant, added, ‘*Last year, I was greatly challenged throughout the summer season and Orthodox and Muslim fasting times since I did not collect and save leftover foods. However, this year I have 165 kilograms of dried leftover food that will be used at the time when I will have difficulty obtaining food in the community*’.

### 3.7 Creating social networks among homeless older people

A crucial coping method used by the research participants was to create a social network among the homeless elderly persons living near the churches. According to the researcher's observations, homeless older adults sit together near the town's churches and converse about various concerns. When different celebrations take place in town, they often went together to beg for food. Furthermore, the homeless older people's social network aids them in reducing emotions of isolation, loneliness and depression. Homeless older adults occasionally assist one another with food and water. Mekonen, a 68‐year‐old homeless man who has been living on the street for 3 years explained:

We, the homeless older adults, usually talk about our lives and other topics. Most of the time, after coming together as homeless older adults in search of food, we have time to chat about our lived experiences. We homeless elderly people who chose to live near churches, in particular, had a strong sense of community. This also greatly aids me in reducing my feelings of loneliness and overcoming the depression that I have been facing on a regular basis. As a result, social networking among us is beneficial to our daily lives.

Homeless elders use their social network as a means of coping with their feelings of loneliness, according to key informant participants from the Labour and Social Affairs office, as well as the leaders of the association serving people with disabilities. They also confirmed that homeless elders who reside at a certain church travel together to beg at rituals, to wash their clothes in the river, and to spend the majority of their time together.

## 4 DISCUSSION

Despite the numerous problems encountered living on the streets, the homeless older people in this study used different coping strategies in an effort to minimise the challenges and meet their survival needs. Begging, holy water, using river water for hygiene and sanitation, living near churches rather than on the street, drying leftover food, and building a social network among homeless older adults were among their coping mechanisms. Rather than waiting for others to come to their rescue, the study participants took the initiative to address the challenges they faced as homeless individuals. Due to their exclusion from the community, homeless elders used varied coping mechanisms as a means of short‐term survival. Begging was a primary approach to getting the resources they needed to meet their daily needs including food, water and clothing. In the absence of public income provision, lacking family support and being considered unemployable, these older adults had few options but to ask for handouts. Likewise, an earlier study by HelpAge International (2011) revealed that homeless older people in Ethiopia used begging as their primary source of income to survive. Consistent with our findings in Ethiopia, older homeless adults in South Africa used a variety of coping mechanisms that included both intrapersonal and interpersonal strategies (Geyer, 2021).

Given the lack of government‐sponsored health services, when the study participants faced illness they paid for their medication through money saved from begging. Thus, the older adults in this study were not passive in reacting to the challenges faced as a homeless person. If they did not have money for medication, they preferred to use holy water to treat their illnesses. Similarly, a study by HelpAge International (2011) indicated that homeless older people in Addis Ababa used a combination of traditional medicine and holy water to manage their health problems.

The findings must be considered in light of the study's limitations including the small non‐random sample and the cross‐sectional nature of the data collection. The findings are based on the input of 10 homeless older adults and four key informants in Kobo Town. Since the study focused on homeless older people who live on the street or around churches and mosques, the findings do not represent other types of homeless older people such as those living with relatives, doubling‐up with neighbours and friends or those who live in institutions. Longitudinal studies and studies with larger samples are needed to document the number of homeless older adults and to substantiate their needs, coping mechanisms and well‐being.

## 5 IMPLICATIONS

Homelessness is the product of a failing social system (Speak, 2020). Despite Ethiopia having established a Plan of Action for Older Persons and Social Protection Policy for Vulnerable Groups, these policies have not been effectively implemented to address the issues of impoverished older adults. Thus, older adults in Ethiopia have no safety net and so are not protected from becoming homeless. Although there are no national policies or programmes being implemented that successfully address the needs of older adults (Kotecho et al., 2022; Zelalem et al., 2021), a small unit––the Elderly Affairs Coordination and Supervision Directorate––was recently set up in Ethiopia's Ministry of Women and Social Affairs. Nevertheless, much work needs to be done to address the needs, as well as the assets, of a growing older population in Ethiopia. This study calls for the development of new policies, as well as changes to current regulations, to address problems and empower homeless older people in the study region and throughout Ethiopia. See Table 1 for a list of recommended action steps and policy innovations.

**TABLE 1:** Recommendations for Addressing the Needs of Homeless Older Adults in Ethiopia

Recognise that older people are a source of wisdom and thus should be practically engaged in developing solutions. Action research on the long‐lasting solution for homeless older people and disadvantaged elders in general is recommended to set solutions in line with the perspective of elders. Providing basic supports geared towards older adults' social protection, health, housing and other social service needs must be viewed in a rights‐based framework. Health extension workers should address the health needs of older adults living on the street. Free and accessible health services should be available to homeless older people. Though older people rely on various coping methods in response to the challenges of living on the street, government intervention is needed to address their challenges. Studies are needed to identify the push factors of older people's migration from rural to urban areas. Since elders migrate from different rural areas of the country, reintegration services should be available to those elders who prefer to return to their rural communities. Working with their biological children and extended family is crucial to solve the problem of those elders for long‐lasting solution. Recognise that social networks among elders on the street are positive and need support from religious leaders and administrators to ensure the psychosocial well‐being of elders until they are reintegrated and/or receive institutional support. Efforts to mobilise resources and create strategies for long‐lasting support should include congregate living options for elders who have no biological children or extended family to support them. Civic associations are important to support elders which need the commitment of the woreda administration by inviting civic associations to prioritise the needs of homeless older adults.

Given the dearth of research on Ethiopia's homeless older adults, further research, including both longitudinal and cross‐sectional studies, are needed to document the extent of homelessness among older adults and to create prevention and intervention strategies as well as bring awareness of older adults' needs. More research is needed to address the risk factors that lead to older persons becoming homeless in Ethiopia, as well as a more thorough investigation of the issues faced by homeless older people and whether those issues vary by gender. Professionals in social work, public health, nursing, medicine, and other fields are needed to contribute to the multidimensional solutions that are needed to assure the well‐being and quality of life of the growing number of older adults in Ethiopia and other Sub‐Saharan nations.

## 6 CONCLUSION

On their own, homeless elders' coping techniques are ineffective in exiting homelessness and ensuring their quality of life. The government of Ethiopia must establish social protection to provide for elders' basic needs, thus helping to prevent homelessness among the growing population of older adults. In the absence of income support and an ageing services infrastructure, the number of homeless older adults will continue to rise. For those older adults who nevertheless end up homeless, a system of support services is needed to ensure their needs are met and their dignity in late life is preserved.

## FUNDING INFORMATION

Addis Ababa University provided funds for the first author to collect the data. Dr. Elias Cheboud and Dr. Abebe Teklu (Ethiopian‐Canadian Professors of Social Work at University of Victoria and Fraser Valley University, British Columbia, Canada) provided additional financial support through their memorial scholarship.

## CONFLICT OF INTEREST

The authors declare that this research and publishing of this article are free of any potential conflict of interest.

## AUTHOR CONTRIBUTIONS

GG and MK conceptualised the study design. GG and MK contributed to data collection and analyses as well as the original drafting of the manuscript. MK provided supervision of data collection and analyses. MA contributed to the literature review, presentation of findings and final drafts of the paper. All authors critically revised the manuscript and accepted its final form.

## Data Availability

Data available on request from the authors
